# Radiological Analysis of Gentamicin Eluting Synthetic Bone Graft Substitute Used in the Management of Patients With Traumatic Bone Voids

**DOI:** 10.7759/cureus.20969

**Published:** 2022-01-05

**Authors:** Ahmed Aljawadi, Thomas Naylor, Amirul Islam, Imad Madhi, Noman Niazi, Mohammed Elmajee, Anand Pillai

**Affiliations:** 1 Trauma and Orthopaedics, Wythenshawe Hospital, Manchester, GBR; 2 Trauma and Orthopaedics, Royal Orthopaedic Hospital NHS Foundation Trust, Birmingham, GBR

**Keywords:** trauma, remodelling, cerament g, bone void, fracture

## Abstract

Background

Management of traumatic bone voids has always been challenging. Gentamicin eluting synthetic bone graft substitute (Cerament-G) showed encouraging results in achieving good bone healing with a satisfactory degree of resorption when utilised as a void filler. This study aims to assess the radiological signs of Cerament-G remodelling when used for patients with traumatic bone voids.

Methods

Retrospective data analysis of all patients admitted to our unit between 2015 and 2021 with traumatic bone voids who had Cerament-G applied intraoperatively as a void filler. Postoperative radiographic images of the fracture site at six weeks, three months, six months, and at the final follow-up were reviewed. The radiological signs of Cerament-G integration, percent of void healing at the final follow-up were assessed.

Results

A total of 51 patients (52 fractures) were included in the study. Among them 10 were female and 41 were male with a mean age of 42.7 (11 - 90) years. The mean void size was 6.58 cm^3^. Mean follow-up duration was 9.73 months. Primary fracture union was achieved in 44 (86.3%) patients. Delayed union was reported in six (11.7%) patients, while one (1.9%) patient had non-union. Twenty-seven (52%) patients had >90% of void healing with normal trabecular bone. Twenty (38.5%) patients had 50-90% void healing with normal bone. Whereas only five (9.5%) patients had less than 50% of void healing.

Conclusion

Cerament-G used as a void filler for patients with traumatic bone void has resulted 98% fracture union rate with good signs of radiological remodelling into a trabecular bone. More than 50% void filling with new trabecular bone was reported in more than 90% of patients. Non-union was reported in only one patient.

## Introduction

Over the years, traumatic bone voids have continued to be a challenge for orthopaedic surgeons. In patients with fractures, bone loss can happen at the time of injury or during surgical debridement of devitalised bone fragments [1,2]. If not managed appropriately, residual voids can subsequently be occupied with fluid (or blood), creating a rich medium for infective microorganisms to flourish and proliferate, increasing the risk of deep infection [3]. Historically, due to difficulties in skeletal reconstruction and potential risk on limb salvage, amputation was the primary treatment for patients with significant traumatic bone loss. However, advancement in techniques of limbs reconstruction has contributed to improving the limb salvage rate in this group of patients [2].

Fractures associated with bone voids had been managed via different strategies, such as autograft, allograft, bone graft substitute, or even by bone shortening [4]. For many years, autologous cancellous bone graft was considered the gold standard, due to its excellent osteoconductive, osteoinductive, and osteogenic properties. However, its limited availability, donor site complications (e.g., pain and bleeding), and increase operative time are the main disadvantages associated with autologous bone graft [5]. On the other hand, allografts have their disadvantages such as altered osteogenic properties due to processing or sterilisation of the graft, as well as the risk of transmitting infections or recipient immunological reaction against the graft [5].

The last few years had witnessed a revolutionary advancement in the properties of bone graft substitutes. Calcium Sulphate had been used for many years as a bone graft substitute. Its availability, cost-effectivity, safety and effectiveness had encouraged many orthopaedic surgeons to use it as a void filler. It was proven to be effective with good bio-absorbability when used to a contained bone void [6].

More recently, Cerament-G was used as a synthetic bone graft substitute and a local antibiotics carrier. Cerament-G is an injectable powder, consists of 40% hydroxyapatite particles in a Calcium Sulphate matrix containing 175 mg gentamicin per 10 ml [7].

In Cerament-G, the osteoconductive properties had been improved by adding hydroxyapatite to the Calcium Sulphate powder. Hydroxyapatite provides a scaffold-like structure as the Calcium Sulphate resorbs, hence it improves bone healing at the fracture site. As well as Cerament-G elutes antibiotics locally which helps to reduce the deep infection rate [8,9]. Cerament-G has been reported to show good radiological signs of successful remodelling and to promote bone healing when used for patients with bone tumours and patients with osteomyelitis [10,11]. This study aims to evaluate the radiological signs of remodelling and the outcomes of bone healing when Cerament-G was used as a void filler and local antibiotics carrier for patients with fractures associated with significant bone voids.

## Materials and methods

Retrospective data collection and analysis was performed of patients admitted between 2015 and 2021 to a single centre with traumatic bone void as a result of fractures. Patients were included in the study if they had Cerament-G applied intraoperatively and if the Cerament-G was radiologically evident on the intraoperative or postoperative check X-ray (XR) images. We excluded patients who didn’t have Cerament-G applied as a void filler, or those who had Cerament-G applied but it was not evident on check XR images.

XR images performed at the time of surgery, at 6-9 weeks postoperatively, at 3 months, at 4-6 months, and at the final follow-up were reviewed. Additionally, computed tomography (CT) scans were performed for some patients when it was difficult to monitor (or confirm) bone healing solely by performing plain XR. These images were reviewed when available.

**Figure 1:**
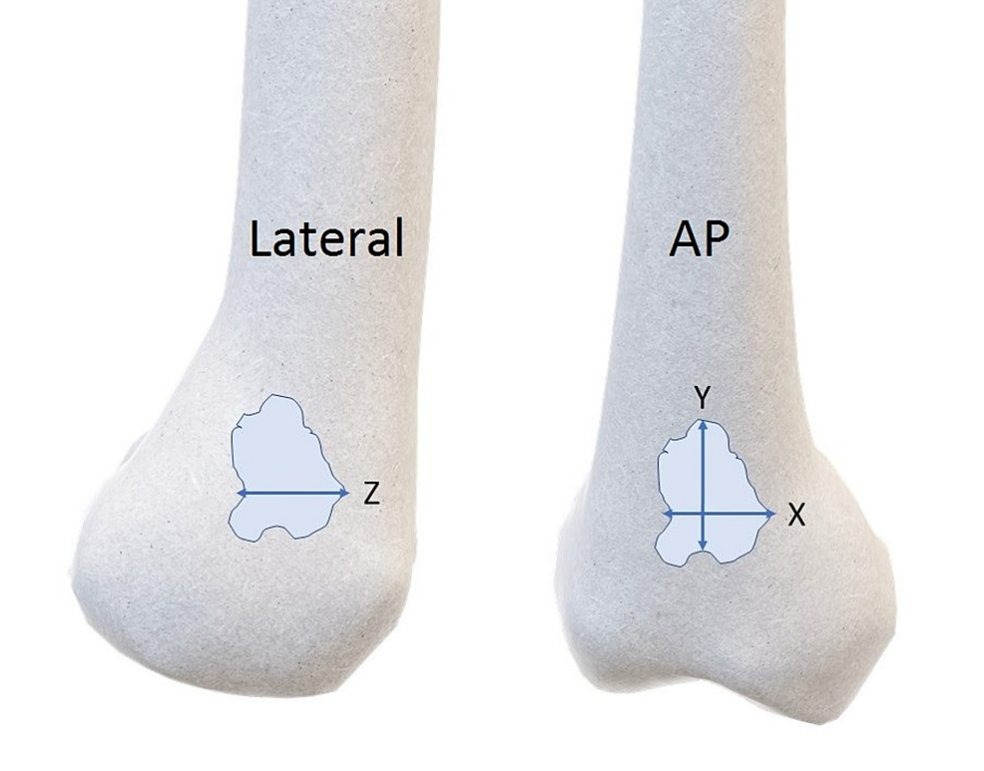
Method of measuring bone void size on X-ray (void size = Y * X * Z cm3).

Perioperative data included patients’ demographics, site of injury, method of fixation, and void size (as assessed on XR images performed at the time of surgery). Voids sizes were only measured after the definitive fixation had been applied to avoid inaccuracies. The void size was concluded by multiplying the gap size on the vertical (Y) and horizontal (X) plane on AP radiographs, and the horizontal (Z) plane on the lateral radiographs (Figure 1). The quality of void filling with Cerament-G intraoperatively was assessed as the following: Good if >90% void filling; average if 50-90% void filling and poor if <50% void filling.

Radiological signs of Cerament-G remodelling on sequential XR images performed postoperatively till final follow-up were assessed. At the final follow-up, modified Neers classification for radiological signs of bone cavity healing was utilised to describe the extent of void healing (Table 1) [12].

**Table 1:** Modified Neers Classification of bone cavity healing

Score	Description
I: Complete healing	Complete healing with normal bone formation with < 1 cm radiolucent area
II: Partial healing	Incomplete bone healing with resorption of < 50% of void filer. Enough cortical healing to reduce risk of further fractures
III: Persistent lesion	> 50% of void filler resorption, with thin cortical healing and potential risk of further fractures
IV: Recurrent lesion	Progression of defect size with new radiolucent area

We have also “quantitatively” assessed for the bone cortical thickness restoration at the fracture site when there was a cortical loss. CT scan images were reviewed when available. Statistical analysis performed using Unpaired (Welch’s) T-Test or Chi-Square test using Windows Microsoft excel 2019 - data analysis pack tool. P-value of < 0.05% considered significant. All tables were produced using Microsoft Word 2019. Figures were designed using Microsoft PowerPoint 2019.

## Results

A total of 51 patients (52 fractures) met our inclusion criteria. There were 10 females and 41 males with a mean age of 42.7 (11 - 90) years. There were 42 (80.7%) open fractures (Gustilo-Anderson type IIIB) and 10 (19.3%) closed fractures. All Gustilo IIIB open fractures were managed by a combined Ortho-Plastic approach. The anatomical area of injury was tibia and fibula in 29 cases, ankle in 10, calcaneal fractures in six patients (one was bilateral), mid-foot in two patients, the femur in two patients, and radius and ulna in two patients. Metaphysis was the site of injury in eight cases, diaphysis in 18, and metaphyseal-diaphyseal junction in 17 cases, as well as there were seven calcaneal fractures and two midfoot fractures. Fracture fixation achieved with plate and screws in 29 patients, circular frame in 12 patients, unilateral external fixation in one patient, combined internal and external fixation in seven patients, IM nail in two patients, K-wires in one patient.

Intraoperative radiological findings

The mean void size was 6.58 cm^3^ (0.25 cm^3^ - 58.6 cm^3^). Good (>90%) intraoperative void filling with Cerament-G was achieved in 39 (75%) patients, average (50 - 90%) void filling in 11 (21.1%) patients, and poor (<50%) void filling in two (3.9%) patients.

Radiological signs of Cerament-G remodelling

**Figure 2:**
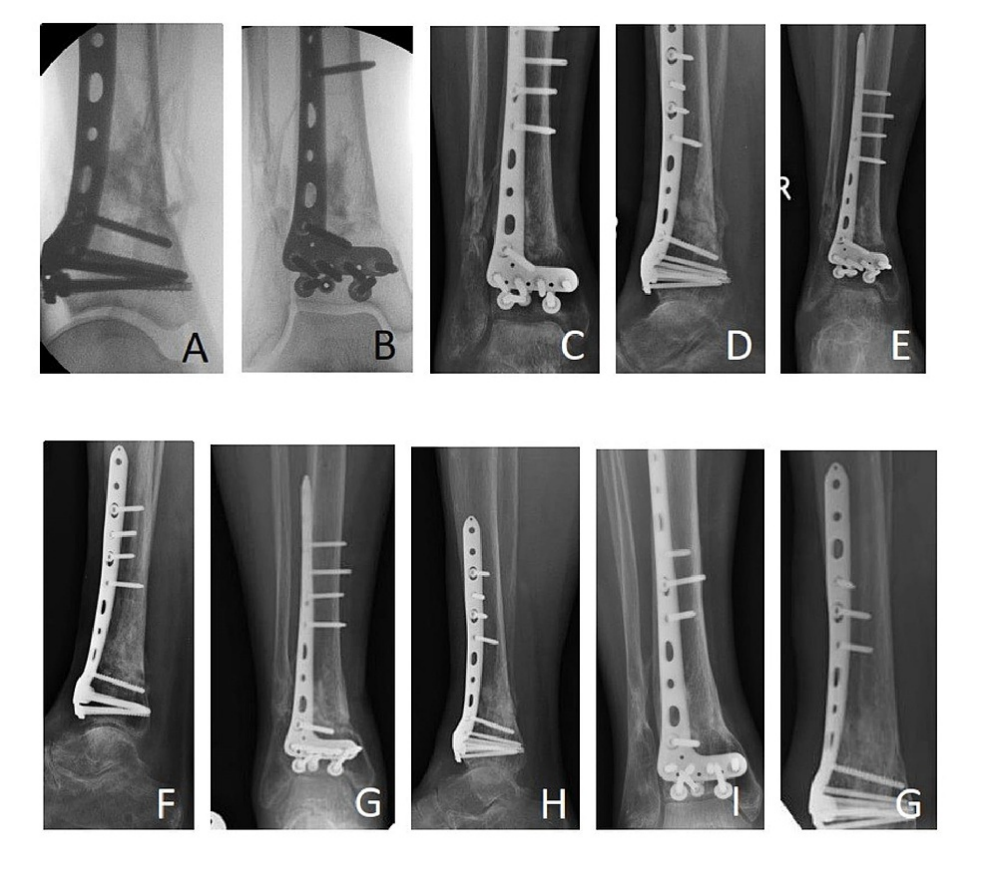
Radiological signs of Cerament-G remodelling seen on X-ray images taken at different stages of healing. A&B: at time of surgery; C&D: at six weeks postoperatively; E&F: at three months postoperatively; G&H: at six months postoperatively; and I&J: at the final follow-up.

Cerament-G showed different radiological signs as it remodelled at different stages of fractures healing. XR images at 6-9 weeks postoperatively were available for 47 (90.3%). XR images performed at that point of time showed partial central resorption or thinning of Cerament-G with occasional Cerement-G leaking. XR at three months was performed for only 35 (67.3%) patients postoperatively, and it showed signs of partial resorption of Cerament-G, with a central area of variable radiodensities surrounded by a relatively radiolucent zone peripherally. While only 39 (75%) patients had XR performed around 4-6 months after surgery, and their images showed signs of a more uniform void, with decreased radiodensity compared to previous XR images. Signs of new trabecular bone formation were occasionally visible (Figure 2).

Radiological findings at the final follow-up

**Figure 3:**
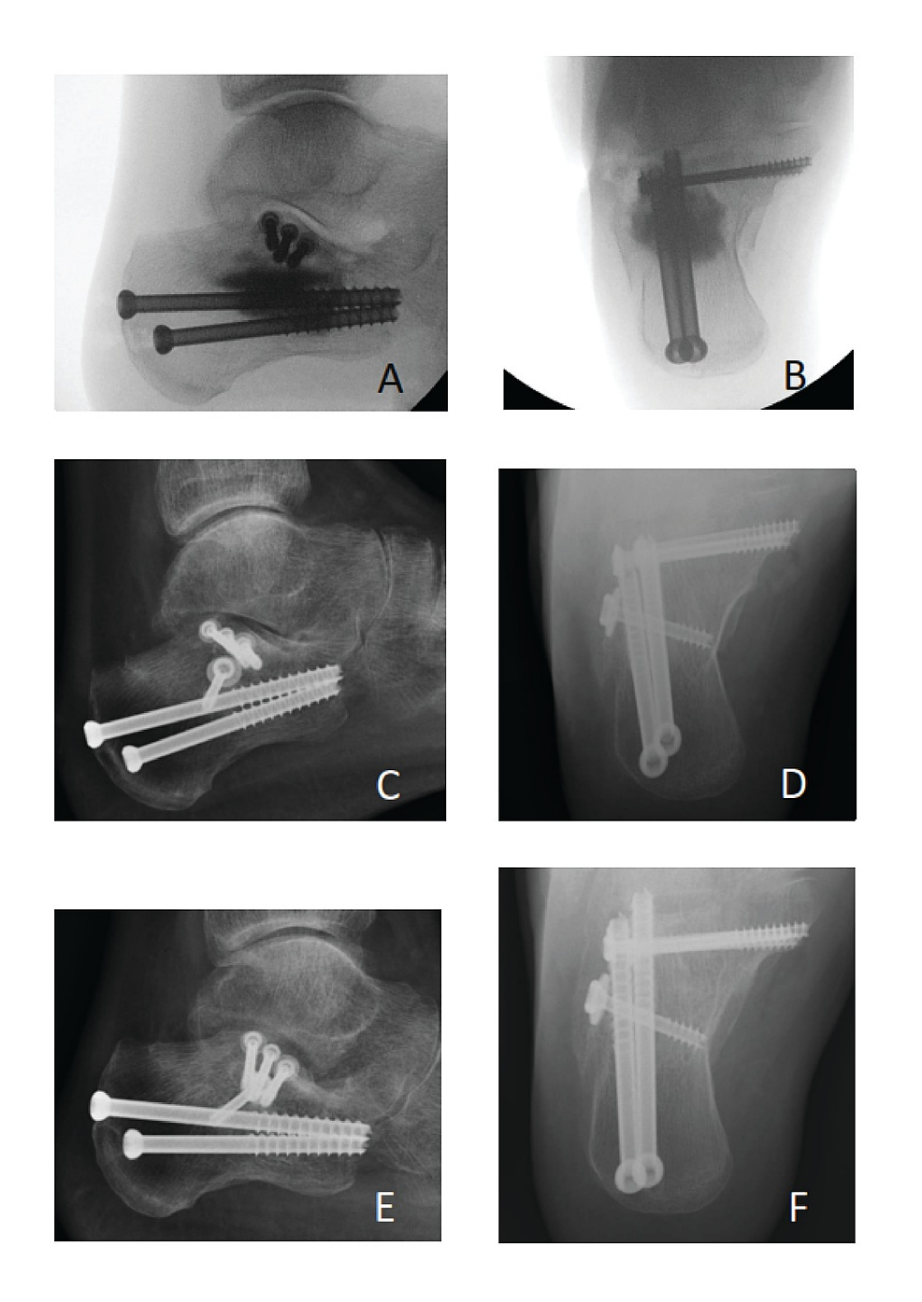
Signs of Cerament-G remodelling in patients with calcaneal fracture. A&B: Fluoroscopic images at the time of surgery; C&D: XR at six weeks postoperatively; E&F: Final XR images at nine weeks postoperatively.

The mean follow-up time was 10 months (2-26 months). One patient had deep infection which required removal of metalwork at six months post-operatively although he missed the subsequent follow-up appointments. This patient was excluded from the total union rate. Primary fracture union was confirmed in 44 (86.28%) cases at the final follow-up. Seven of these 44 patients had calcaneal fractures and successful fracture healing was confirmed clinically and radiologically at 2.57 (2-4) months postoperatively (Figure 3).

Six (11.76%) patients had delayed union, as evidenced by minimal radiological signs of bone healing at nine months postoperatively. They had Bone Marrow Aspirate Concentrate (BMAC) injected into the fracture site. Bone healing was confirmed for these patients at 6.7 months following the BMAC. One (1.96%) patient had non-union with no signs of bone healing at 12 months after surgery, yet this patient didn’t attend any further follow-up appointment afterwards. This patient was 20 years old, had midshaft Tibia and Fibula Gustilo IIIB open fracture, and had no significant past medical history.

Fracture healing time varied depending on the anatomical area of injury. The mean healing time for the seven calcaneal fractures (2.5 months) and the 2 feet fractures (4 months) was significantly shorter compared to the other 43 patients with long bone fractures (which was 11 months) (Table 2). Regarding patients with long bone fractures, the fracture was described as per its anatomical location within the bone into metaphyseal, diaphyseal or combined metaphyseal and diaphyseal lesions. Bone healing rate in each one of these three areas was assessed and compared to the healing rates in the other two areas. The healing time was almost identical in metaphyseal part of the bone compared to diaphyseal or combined lesions with no statistically significant difference (Table 2).

**Table 2:** Healing time and rate of bone voids filling in different anatomical sites.

		Long bones fractures								Foot fractures				
		Metaphysis			Diaphysis		Combined			Calcaneum		Mid-foot		Total
		proximal	distal	P-Value		P-Value	proximal	distal	P-Value		P-Value		P-Value	
Union Time (Months)			11	0.988559	10.5	0.653741	12	11.5	0.65021	2.5	3.58023E-12	4	0.013246	
Percent of Void Healing	>90%		4 (50%)	0.941863	7 (38.8%)	0.268129	1 (50%)	9 (60%)	0.289456	4 (57%)	0.68359	2 (100%)	0.15709	
	50-90%		2 (25%)		9 (50%)		0	6 (40%)		3 (43%)		0		
	< 50%		2 (25%)		2 (11.2%)		1 (50%)	0		0		0		
Total Number of Cases		0	8		18		2	15		7		2		52

Patients’ age did not have a significant effect on fracture healing for patients with long bone injuries. There were 28 patients younger than 50 years old and 15 patients older than 50 years of age at the time of their injuries. Mean fracture healing time for patients younger than 50 years old was 10.7 months compared to 11.4 months for those older than 50 years old (P-value: 0.68368).

Radiological signs of void healing on plain XR images

At the final follow-up, XR images showed that 27 (52%) patients had > 90% of void healing with new trabecular bone (Neers type I). Twenty (38.5%) patients had 50-90% void healing with new bone (Neers type II). Whereas only five (9.5%) patients had less than 50% of void healing (Neers type III). Results showed mild variation in void healing between different anatomical areas with no statistically significant difference. There was > 90% void healing in 57% of calcaneal fractures, compared to 48.8% of those patients who had long bones fractures. Within the same context, there was > 90% void healing in 50% of patients with metaphyseal voids, compared to 38.8% of those with diaphyseal voids, and 58.8% of combined voids (Table 2). Table 3 summarises the percent of void healing for patients with open fractures compared to those with closed injuries.

**Table 3:** Void size and percent of void healing at the final follow-up for patients with open and closed injuries.

	Age	Void size (cm)	> 90% void filling (%)	50-90% Void filling (%)	< 50% void filling (%)
Open fractures	42.15	7.3	22 (52.4%)	15 (35.7%)	5 (11.9%)
Closed fractures	45.1	5.3	5 (50%)	5 (50%)	0

On the other hand, there was a cortical defect at the site of fracture in 39 (75%) patients. For these patients, > 90% of cortical thickness was restored in 20 (51.3%) at the final check XR images. Thirteen (33.3%) had 50-90% restoration of cortical thickness. While cortex remained less than 50% thick in the remaining six (15.4%) patients as compared to the surrounding normal bone.

Radiological finding on CT scan images

**Figure 4:**
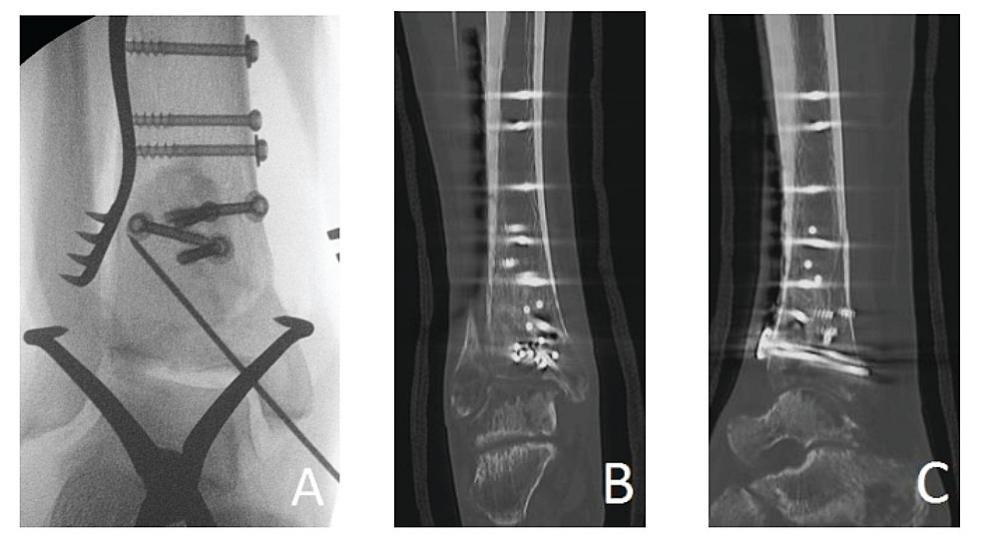
Signs of Cerament-G remodelling as seen in CT scan. A: Cerament-G as a void filler intraoperatively; B: CT scan coronal view at six months postoperatively; C: CT scan sagittal view at six months postoperatively.

CT scan was performed for 26 (50%) patients postoperatively, however, two patients had their scan performed within one week after surgery and they were excluded. This resulted in CT scan images being available for only 24 (46.1%) patients. The mean time from surgery till the last CT scan was 8.3 months (1-24 months). More than 90% void filling with normal bone was confirmed on CT scan for five patients (20.8%). Fourteen (58.4%) patients had 50-90% void filling, and only five (20.8%) patients had < 50% void filling. CT scan images showed that bone cortex at fracture site was restored up to 90% of its thickness in four (16.7%) patients. Bone cortex was restored up to 50-90% in 11 (45.8%) patients, whereas less than 50% of the cortex was restored in nine (37.5%) patients. Figure 4 shows the CT scan images of Cerament-G remodelling at six months postoperatively.

## Discussion

Many authors reported satisfactory outcomes with Cerament-G as a bone void filler in promoting bone healing and trabecular bone formation [13]. In an animal study performed by Dvorzhinskiy et al. in 2015, they concluded that Cerament-G had resulted in a 56% increase in bone formation at the void site when it was applied as a void filler compared to cases where no void filler was applied [14]. Iundusi et al. had investigated the outcomes of using Cerament-G as a bone graft substitute for patients with depressed tibial plateau fractures. The results showed that Cerament-G helped to maintain the fracture reduction within acceptable limits until fracture healing was confirmed, Cerament-G resorbed within eight months postoperatively with successful fracture healing [15]. More recently, Ferguson et al. in 2019 had analysed the radiological and histological signs of Cerament-G remodelling when used for patients with chronic osteomyelitis [11]. In their study, Ferguson et al. reviewed 163 patients with chronic osteomyelitis where patients had excision of the unhealthy bone and Cerament-G implemented as a void filler. After 20 months of follow-up, infection was eradicated in 95% of cases. Mean void healing was 73.8% at the final follow-up with a significantly higher void healing rate in metaphyseal compared to diaphyseal fractures [11].

All patients included in our study had a traumatic bone void, and 80.7% of them had Gustilo IIIB open fractures. Gustilo IIIB open fractures are severe injuries and are usually associated with a high rate of morbidities and mortality [16,17]. In patients with open fractures and bone void, the soft tissue trauma and/or loss would result in a suboptimal environment for bone healing and will increase the risks of complications, including infection [4,18]. Nonetheless, the results from our study showed that more than half of patients had more than 90% void healing with new trabecular bone and significant resorption of Cerament-G at the end of their follow-up. On the other hand, only five patients had less than 50% of void filling. Having an open fracture didn’t affect void healing rate compared to patients with closed injuries, as 50% of patients with closed injuries had > 90% void healing compared to 52.4% of patients with open fractures. In fact, the mean void size for patients with open fractures was bigger (7.3 cm) compared to patients with closed fractures (5.3 cm).

Void healing

Average healing time was significantly shorter for patients with calcaneal (2.5 months) or feet (four months) injures compared to those with long bones injuries (11 months) whereas no statistically significant difference in healing time was identified between metaphyseal bone voids (11 months) compared to diaphyseal (10.5 months) or combined voids (11.5 months). On the other hand, void filling rate has varied between different anatomical areas with no statistically significant correlation (Table 2). The excellent bone remodelling seen in cancellous bone voids (calcaneal and foot injuries) in our study would suggest that vascularity plays a significant role in bone healing. This may be particularly important in injuries with vascular compromise, severe soft tissue loss or periosteal stripping.

Deep infection

Deep metal work infection was reported in one patient at six months after surgery. This patient was 30 years old presented with a crush injury to his leg. His initial surgery involved open reduction and internal fixation (ORIF) + free antero-lateral thigh (ALT) flap. He did not have any significant past medical history. Metalwork was removed at six months postoperatively due to deep collection, however, this patient did not attend for follow-up after his revision surgery.

Cerament-G remodelling

Different stages of fracture healing showed different radiological signs of Cerament-G remodelling due to its resorption. This progressed from partial radiolucency seen at 4-6 weeks postoperatively toward more uniform healing seen at around six months postoperatively. Ferguson et al. had described three radiological signs of Cerament-G remodelling seen subsequently on XR images: Halo sign was seen at six weeks postoperatively, Marble sign at around three months postoperatively and Puddle sign at around six months postoperatively (Table 4) [11].

**Table 4:** Radiological signs of Cerament-G remodelling as described by Ferguson et al.

Sign	Timing	Description
Halo sign	6 weeks	Peripheral radio-dense ring
Marble sign	3 months	Marble shaped remnant of the Cerament-G
Puddle sign	6 months	Radio-dense area at the bottom of the lesion

Nonetheless, we were unable to identify these signs clearly on patients' XR images in the current series. This can be explained by the fact that all patients in our study presented after trauma with extremely irregular bone void and/or cortical disruption while patients included in Ferguson’s series were presented after osteomyelitis where a more regularly shaped and contained bone void would be expected.

Findings on CT scan images

The CT scan findings of Cerament remodelling were investigated by Marcia et al. who reviewed the CT scan findings of 66 patients who had Cerament injected as a void filler following vertebral fractures. At one-year follow-up, their results showed that Cerament had been successfully remodelled and incorporated into the vertebral bodies [19]. In our series, CT scan images were available for only 24 patients. However, these scans were performed for some patients before the fracture union was confirmed, thus it showed lower figures in terms of fracture healing compared to the XR images at the final follow-up. The result of these scans showed that more than 79% of patients had more than 50% void healing with new trabecular bone.

## Conclusions

Cerament-G used for patients with traumatic bone void has resulted in 98% fracture union rate with good signs of radiological remodelling of Cerement-G into a trabecular bone. More than 50% void filling with new trabecular bone was reported in around 90% of patients. Similarly, more than 50% of normal cortical thickness was restored in 85% of patients at the final follow-up who initially had cortical disruption.
